# Understanding the Compatibility of Fluoride-Based Radiopharmaceutical Reaction Solutions and PDMS

**DOI:** 10.1021/acsami.5c21729

**Published:** 2025-12-22

**Authors:** Mark Mc Veigh, Charles Frech, Mai Lin, Robert Ta, H. Charles Manning, Leon M. Bellan

**Affiliations:** 1 Interdisciplinary Materials Science, 5718 Vanderbilt University, Nashville, Tennessee 37235, United States; 2 Department of Biomedical Engineering, 5718 Vanderbilt University, Nashville, Tennessee 37235, United States; 3 Cyclotron Radiochemistry Facility, 4002 The University of Texas MD Anderson Cancer Center, Houston, Texas 77054, United States; 4 RADIATE R&D Platform, 4002 The University of Texas MD Anderson Cancer Center, Houston, Texas 77054, United States; 5 Department of Nuclear Medicine, 4002 The University of Texas MD Anderson Cancer Center, Houston, Texas 77030, United States; 6 Department of Mechanical Engineering, 5718 Vanderbilt University, Nashville, Tennessee 37235, United States

**Keywords:** radiopharmaceuticals, microfluidics, PDMS, fluoride, compatibility

## Abstract

Microfluidic devices offer unique and exciting benefits when applied to radiopharmaceutical manufacturing, and these platforms are now starting to be integrated into commercial products. The field has strayed away from the use of polydimethylsiloxane (PDMS), the most common microfluidic device material, due to its suspected incompatibility with ^18^F, the most commonly used radionuclide. However, existing literature provides conflicting conclusions as to the existence and extent of this incompatibility. In this study, we use several analytical instruments to uncover the underlying interaction between fluoride and PDMS. SEM imaging and profilometry confirm the reactive relationship between the two materials and suggest that this interaction only occurs when the reaction solution is fully evaporated and crystallized salts are in contact with PDMS. Furthermore, GC-MS identifies fluoride-containing volatile species that can account for loss of fluoride in previous studies and additionally reveals an incompatibility between PDMS and K_2_CO_3_ (a commonly used component of radiofluorination reaction solutions). These results confirm the need for microfluidic radiofluorination devices to avoid the use of PDMS in most contexts but may allow for inexpensive design and testing of liquid state operations (such as concentration, purification, and mixing) using the material.

## Graphical abstract

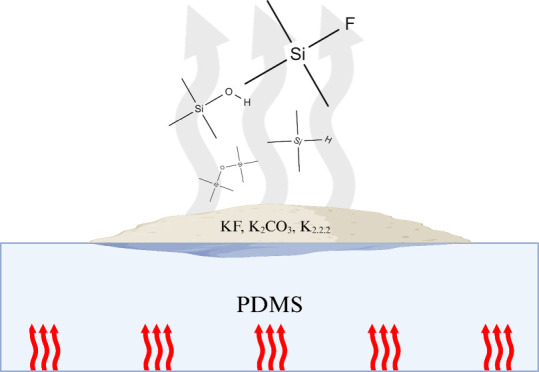

## Introduction

In pursuit of dose-on-demand manufacturing of radiopharmaceuticals, significant effort has been invested into developing microfluidic radiotracer synthesis units. Many designs have used polydimethylsiloxane (PDMS),^1–5^ the most common material used to fabricate microfluidic devices. PDMS is a well-studied, low-cost, and easily employed material routinely used in the field of microfluidics but has seen a drop in use as a material for radiopharmaceutical applications due to its suspected incompatibility with fluoride. ^18^F is the most common radionuclide used in positron emission tomography (PET), accounting for 65% of approved PET radiotracers^6^ and well over 95% of scans due to the ubiquitous use of [^18^F]­fluorodeoxyglucose ([^18^F]­FDG).^7^ Thus, any incompatibility that reduces the reaction efficiency or overall yield could result in large amounts of waste, negating the potential benefits of microfluidic approaches. Additionally, if unwanted byproducts are created due to side reactions, there may be significant good manufacturing practice (GMP) concerns. While many studies in this area are exploring the use of other materials to produce such devices, several papers have been published citing notably varying levels of incompatibility with PDMS. To ensure that all current and future researchers considering PDMS for any part of a radiofluorination or similar process are fully aware of any incompatibilities, it is important to establish a concrete understanding of any interactions between PDMS and the reagents employed for ^18^F-based radiosynthesis.

In 2010, Elizarov et al. published one of the earlier and most influential studies in the field of microfluidic synthesis of radiotracers. The study, which used a PDMS device, reported losses of fluoride of up to 95%. The authors hypothesized that fluoride reacts with and etches PDMS, forming volatile species. These losses were specifically noted to occur during evaporation steps that required prolonged heating.^1^ Later that same year, Tseng et al. released a study focused on this problem by measuring the amount of activity lost during evaporation. Results showed minimal loss of activity during evaporation (while there was still solvent present) but a significant loss when the heating time was extended beyond complete evaporation. Again, this loss was hypothesized to be due to fluoride reacting with PDMS to create volatile species.^8^ These papers suggest that fluoride and PDMS readily react, but data identifying or confirming the presence of volatile species or suggesting a reaction mechanism are not provided. Outside the radiopharmaceutical field, fluoride has been reported as an effective PDMS etchant for wet etching via tetrabutylammonium fluoride (TBAF)^9,10^ and dry etching via SF_6_ or CF_4_.^11,12^

Although the literature from both within and outside the microfluidic radiopharmaceutical community suggests that fluoride is reactive with PDMS, multiple sources have come to seemingly contradictory conclusions. In 2018, Cesaria et al. ran a series of studies testing the compatibility of PDMS and chemicals (including fluoride) associated with the synthesis of [^18^F]­F-DOPA. The authors soaked PDMS slabs in an aqueous fluoride solution and analyzed the surface of the soaked slabs with X-ray photoelectron spectroscopy (XPS). Their data suggested that fluoride was being incorporated onto the PDMS surface, specifically through C–F bonds, but with sufficient washing with distilled water, the fluoride could be completely washed off.^13^ The following year, Fernandez-Maza et al. released a study in which microreactors were filled with ^18^F-containing solutions, heated to evaporate the solution, and subsequently washed with 1 or 2 mL of water. Measurements of radioactivity in the reactor were taken before evaporation, after evaporation, and after washing suggested that less than 1% of activity was lost to the PDMS. From this, the authors concluded that fluoride does not interact with PDMS under conditions relevant to radiosynthesis.^14^

Based on concerns suggested in earlier reports, many researchers in the radiosynthesis field have shifted away from the use of PDMS and instead have relied on more chemically inert materials such as cyclic olefin polymers/copolymers (COP/COC)^15,16^ and polyether ether ketone (PEEK).^17^ These replacement materials are more expensive and difficult to work with, while PDMS remains a popular material for microfluidic systems. Therefore, establishing a fundamental understanding of any incompatibility will determine to what degree PDMS can or cannot be used for large-scale production, prototyping, valving, etc.

Previous studies investigating potential compatibility issues have mostly relied on measurements of radioactivity at various locations on- and off- chip, but these results do not provide insight into the underlying mechanism. Therefore, this study aims to provide data to directly address this interaction on a chemical level and provide researchers with a complete understanding of any incompatibility. We report data confirming the reactive nature of fluoride and PDMS, highlight the additional incompatibility with K_2_CO_3_, and propose a mechanism that reconciles the results of previously published, seemingly conflicting, experimental results.

## Materials and Methods

### Reagents

Cold (nonradioactive) ^19^F was used in these studies as opposed to radioactive ^18^F to enable the use of a wider range of analytical equipment and directly probe the underlying mechanisms of the interactions between fluoride and PDMS. Due to their matching electronic structure, the reactive nature of the two species should be near identical.^18,19^

Potassium fluoride (KF), Kryptofix 2.2.2 (K_2.2.2_), potassium carbonate (K_2_CO_3_), and anhydrous acetonitrile (ACN) were purchased from Sigma-Aldrich. Polydimethylsiloxane (PDMS) elastomer and curing agent (SYLGARD 184) were purchased from Ellsworth Adhesives.

Reaction solutions were formulated based on standard radiofluorination conditions used for production of radiotracers at MD Anderson Cancer Center. These conditions call for each mL of reaction solution to contain 3 mg of K_2_CO_3_ and 10 mg of K_2.2.2_ with varying amounts of fluoride. For a 1 mL, single dose run containing 100 mCi (3.7 GBq) of radioactivity, fluoride (with a molar activity of 6.03 × 10^14^ GBq/mol) would have a final concentration of 6.15 pM. This concentration was undetectable by analytical tools, so for this study, the amount of fluoride was increased (while holding concentrations of both K_2_CO_3_ and K_2.2.2_ constant) until sufficient signal was achieved (S1). The final solution used for analysis contained 61.5 mM KF, 26.2 mM K_2.2.2_, and 21.7 mM K_2_CO_3_ in 80:20 (v:v) ACN:H_2_O (referred to as 10^10^x_F). By straying from standard radiosynthesis conditions and using nonradioactive ^19^F and increased concentrations of KF, the reaction kinetics may be different, but the mechanism should be the same and allow for valuable insights into the viability of using PDMS in the presence of radiofluorination reactions.

### PDMS Slab Fabrication

Throughout all experiments, PDMS (Dow SYLGARD 184, Ellsworth Adhesives) was cast using a 10:1 ratio of base elastomer to curing agent. The components were mixed thoroughly using a THINKY mixer, poured into an experiment-specific mold, and degassed for 30 min. The PDMS was then cured at 80 °C in an oven overnight.

### Scanning Electron Microscopy-Energy Dispersive X-ray Spectroscopy

For scanning electron microscopy-energy dispersive X-ray spectroscopy (SEM-EDS) experiments, sufficient PDMS was poured into 60 mm Petri dishes to produce a roughly 3 mm thick slab. Once cured, the slab was placed at the center of a preheated 105 °C hot plate shortly before solution deposition. A 50 μL aliquot of 10^10^x_F was deposited at the center of each mold, and the time of deposition was recorded. The slab was removed from the hot plate after 20 min and allowed to cool in a small Petri dish. Fresh, uncured PDMS was then poured over the slab to embed the crystallized salt in PDMS. The PDMS was allowed to cure at room temperature for 2 days. Once cured, a razor blade was used to cut out a small section of PDMS, revealing a cross-sectional area of the interface between PDMS and crystallized salt. The sections were coated in a thin layer of gold and imaged using a Zeiss Merlin SEM equipped with an Oxford X-MAX 50 SDD EDS system. EDS was completed using 15 kV column voltage and 1 nA probe current.

### Profilometry

Just as for the SEM experiments, 60 mm Petri dishes were used to create slabs of PDMS. Each slab was placed at the center of a preheated 105 °C hot plate shortly before solution deposition. A 50 μL aliquot of 10^10^x_F was deposited at the center of each slab, and the time of deposition was recorded. The slabs were removed from the hot plate at varying time points following deposition. After removal, each device was rinsed with 25 mL of ACN to dissolve and remove the crystallized salt from the surface. To identify the presence and extent of any etching, surface topography measurements at the deposition site were collected using a Bruker Dektak 150 stylus profilometer. Each 2 min scan was 10 mm in length and used a 2.5 μm stylus tip radius with a stylus force of 1 mg (9.8 μN). Scans were leveled using a quadratic fit line^20^ to remove baseline tilt and curvature (due to any possible warping).

### Gas Chromatography Mass Spectroscopy

Roughly 0.4 g of PDMS was poured into 2 mL glass vials, degassed, and cured overnight. A sand bath was preheated to 105 °C. 50 μL of various solutions was deposited into individual vials and placed into the sand bath for 1 h with the vial cap removed. A sand bath and 1 h of heating were required to complete evaporation in a reasonable amount of time due to limited mass transport out of the vial. After 1 h had elapsed, the vials were capped and heated for an additional 20 min. The vials were then removed from the sand bath and transferred to the Vanderbilt Mass Spectrometry Research Center, and using a 500 μL Hamilton SampleLock syringe, a 100 μL sample of the headspace directly above the salt residue was collected. The samples were then analyzed using an Agilent 5973 single quadrupole GC-MS system.

## Results and Discussion

### Elemental Analysis

To determine if fluoride adheres to the surface of PDMS as previously suggested^13^ or diffuses into the porous structure, SEM-EDS imaging was used to spatially analyze the location of fluorine in relation to the surface of a PDMS slab.

Based on the elemental map of the cross section of the interface between the salt residue and PDMS (Figure 1C), fluoride does not diffuse into PDMS in appreciable amounts. Instead, there is a sharp reduction in the fluoride concentration between salt and PDMS (Figure 1E). Furthermore, additional tests have shown that with excessive washing (beyond what would be feasible in a standard radiopharmaceutical manufacturing run), almost all salt can be washed away (S2). These data sets complement each other to suggest that fluoride is not being retained by the PDMS matrix. The ability to wash off fluoride and leave behind minimal residue aligns with the findings of previous reports.^8,13,14^ Still, SEM imaging suggests significant surface damage after evaporation (S2), indicating a potentially reactive relationship between PDMS and 10^10^x_F.

**Figure 1:**
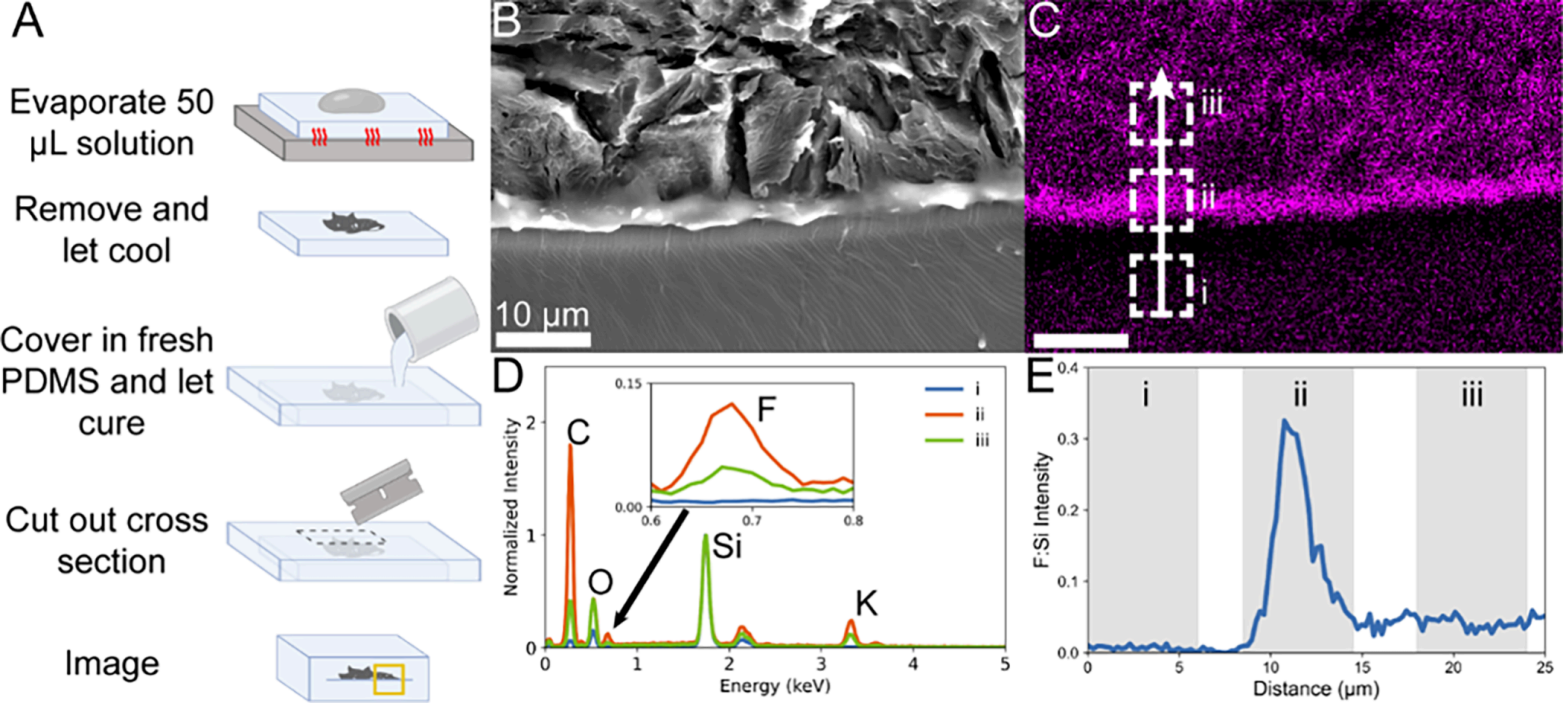
(A) 50 μL of 10^10^x_F was evaporated on a PDMS slab, which was subsequently covered with additional PDMS. After this additional PDMS was allowed to cure, the full structure was sliced with a blade to expose a cross section of the salt–PDMS interface. This interface was (B) imaged using SEM and (C) analyzed using EDS. EDS data was used to collect (D) the spectra of (i) the bulk PDMS, (ii) the salt–PDMS interface, and (iii) crystallized salt flakes. Each spectrum was normalized to its Si peak. Furthermore, (E) a line scan covering each of these areas of interest was collected and normalized to Si. The EDS image and line scan were processed using a binning factor of 4 to increase the signal-to-noise ratio.

### Surface Analysis

The combination of SEM-EDS results indicating a surface-based interaction and previous reports suggesting a reaction between PDMS and fluoride yielding volatile products^1,8^ drove our hypothesis that the PDMS was being etched away. Profilometry was used to measure the PDMS surface at different time points to visualize the progression of etching (Figure 2).

**Figure 2:**
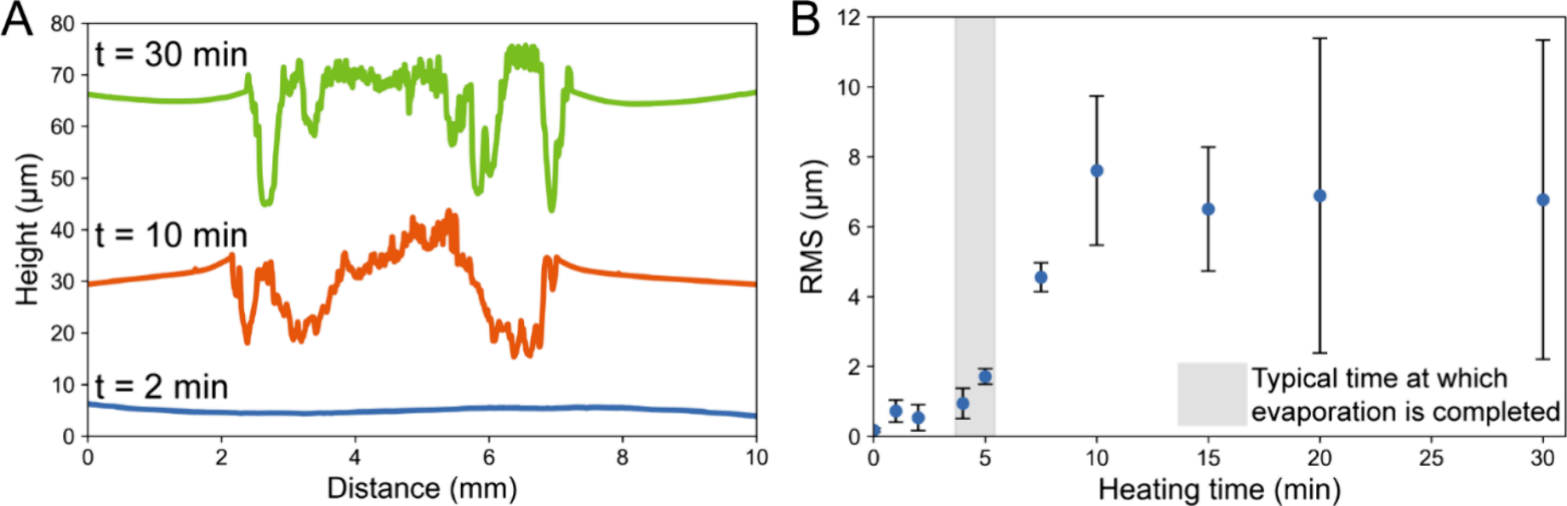
(A) Profilometry results showing the height as a function of distance across the surface of PDMS slabs exposed to 10^10^x_F heated for 2, 10, and 30 min from the time of deposition. Scans were offset by 5, 30, and 65 μm to display on a single plot. Samples that were removed from heat before evaporation was complete were smooth with no detectable damage to the surface. Meanwhile, samples heated beyond the time of complete evaporation contained deep valleys measuring 20 μm or more, indicating significant etching. (B) Root mean square (RMS) roughness values (*n* = 3; error bars indicate standard deviation) show a clear increase in roughness after evaporation is complete, indicating that etching occurs when crystallized salts are heated on the PDMS surface.

Profilometry shows clear patterns of significant etching upon extended exposure to heat. Interestingly, no etching is observed if the reaction solution has not fully evaporated. In other words, the PDMS does not seem to be damaged due to exposure to heated solutions but rather due to exposure to heated crystallized salts. Etching was most severe toward the edges of the contact region where, likely due to the coffee ring effect, solutes were driven to the edge of the evaporating droplet, leading to more concentrated areas of salt. The reaction only occurring in the salt phase is consistent with the findings of Tseng et al., which indicated that the activity within a channel drops dramatically only after evaporation and with extended heating.^8^ The etching of PDMS combined with the lack of fluoride after washing, as seen through SEM-EDS (S2), strongly supports the idea that the loss of fluoride seen in previous studies is due to it reacting with PDMS to create volatile compounds.

### Reaction Mechanism

Although previous reports have suggested the production of volatile compounds, a mechanism by which this occurs has not been proposed. Direct detection of volatile products via GC-MS headspace sampling (instead of the indirect measurement of activity loss used in previous reports) provides a more conclusive understanding of the underlying chemistry.

To isolate the components that play the most important role in damaging the PDMS surface, several different solutions (all using the same 80:20 (v:v) ACN:H_2_O solvent system) were evaporated from a PDMS surface and the headspace analyzed using GC-MS. The resultant spectra show the clear production of volatile substances by some of these solutions. A solution of only K_2.2.2_ does not show significant production of any volatile species (Figure 3A i,ii), but when either K_2_CO_3_ (Figure 3A iii,iv) or KF (Figure 3A v,vi) were introduced, several PDMS degradation products were detected. The inclusion of K_2_CO_3_ yielded several PDMS degradation products, including trimethylsilane (Figure 3B), trimethylsilanol (Figure 3C), and hexamethyldisiloxane (Figure 3E). When fluoride was introduced to the solution, trimethylfluorosilane (Figure 3D) was produced. Evidently, both fluoride and K_2_CO_3_ react with PDMS to create various volatile species. Additional profilometry experiments with decreasing concentrations of KF show that etching remains relatively constant (S3), suggesting that K_2_CO_3_ is the main component causing PDMS breakdown. The production of F-containing volatile species clearly identifies a major source of potential activity loss when working with radioactive fluoride.

**Figure 3:**
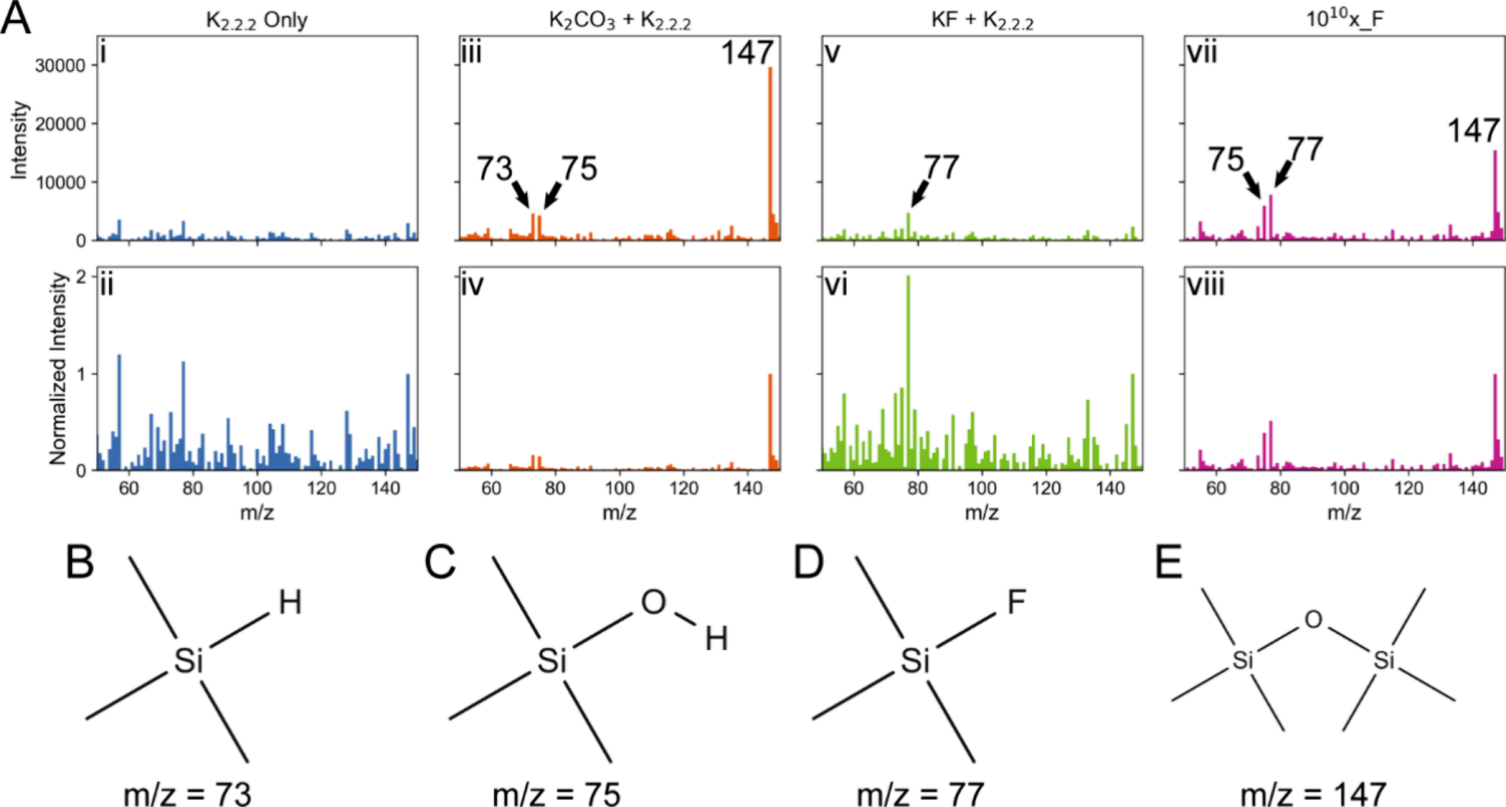
(A) MS spectra from RT = 1.4 min of solutions containing (i,ii) only K_2.2.2_, (iii,iv) K_2_CO_3_ and (K_2.2.2_, (v,vi) KF and K_2.2.2_, and (vii,viii) 10^10^x_F. All solutions were made in 80:20 (v:v) ACN:H_2_O, and the concentrations of each component in the three partial solutions were identical to their respective concentration in 10^10^x_F (e.g., the concentration of KF was 61.5 mM). Each normalized spectrum was normalized to its *m*/*z* = 147 peak. Dominant products of the reactions include (B) trimethylsilane, (C) trimethylsilanol, (D) trimethylfluorosilane, and (E) hexamethyldisiloxane.

The Si–O bond is highly polarized due to the large difference in electronegativity (Si = 1.74 and O = 3.50). In fact, the bond is polarized to the point of being considered an intermediate bond with ionic properties, resulting in a significant partial positive charge on the Si atoms.^21^ The partial positive charge makes the Si susceptible to attacks from nucleophiles, in this case, both F^–^ and CO_3_^2–^. F^–^ acts as the nucleophile by directly attacking the positively charged Si, cleaving the Si–O bond.^10^ It then bonds with silicon to form Si–F, an incredibly strong bond, creating trimethylfluorosilane (Figure 3D). We suspect that the CO_3_^2–^ also cleaves the Si–O bond through the negatively charged oxygen atoms of CO_3_^2–^ attacking the positively charged Si.^22^ In both cases, when fluoride is not present to bind to, the cleaved sections of PDMS react with available hydrogen and hydroxyl groups to create trimethylsilane (Figure 3B) and trimethylsilanol (Figure 3C) or with other cleaved sections to create low molecular weight compounds such as hexamethyldisiloxane (Figure 3E).

## Conclusions

Using SEM-EDS, profilometry, and GC-MS, the interaction of PDMS and a fluoride-based radiotracer synthesis solution was explored. Our results provide a more complete picture of the interaction, supported not only by the data presented in this study but also by the results of previous reports. In solution, fluoride and carbonate exhibit minimal interaction with PDMS and can be washed off (albeit with volumes beyond those reasonable for microfluidic radiopharmaceutical production) as also concluded by Cesaria et al.^13^ Using this fact, if removal from heat is timed well with evaporation, fluoride loss and PDMS damage may be minimized, as likely occurred in the study by Fernandez-Maza et al.^14^ However, if heating is extended beyond the point of complete evaporation, then extensive degradation of the PDMS will occur by both F^–^ and CO_3_^–2^, creating volatile products.

With improved knowledge of this interaction, researchers developing microfluidic radiosynthesis platforms can make more informed decisions about material choice. These results conclusively show that when used for evaporation, PDMS and radiofluorination solutions are highly reactive, and their combined use should be avoided. Still, the results of this and previous work suggest that PDMS may be used for liquid state operations, which may provide avenues for fast, inexpensive iteration of designs of secondary components such as exchange columns or mixing channels. As this field of research continues toward commercialization, cost analysis in development is critical, and knowing the exact limitations of all involved materials will allow for more effective prototyping and production.

## Supplementary Material



## Data Availability

The data underlying this study are openly available in Zenodo at https://doi.org/10.5281/zenodo.17822119

## Notes

Additional experimental data including SEM-EDS analysis of increasing KF concentrations, SEM-EDS analysis of (un)­washed slabs, and profilometry analysis of increasing KF concentrations (PDF)
