# Supplementary material for: Understanding the Compatibility of Fluoride-Based Radiopharmaceutical Reaction Solutions and PDMS
Source: ACS Appl Mater Interfaces. 2025 Dec 22;18(1):2775–80. doi: 10.1021/acsami.5c21729 (PMC12781053; doi:10.1021/acsami.5c21729)
Supplement: Supplementary file 1 [file am5c21729_si_001.pdf]

Supporting Information

Understanding the Compatibility of Fluoride-Based Radiopharmaceutical Reaction Solutions and PDMS

*Mark Mc Veigh[1], Charles Frech[2], Mai Lin[3,4], Robert Ta[3], H. Charles Manning[3,4,5], Leon M. Bellan[1,2,6]**

[1]Interdisciplinary Materials Science, Vanderbilt University, Nashville, Tennessee 37235, USA

[2]Department of Biomedical Engineering, Vanderbilt University, Nashville, Tennessee 37235, USA

[3]Cyclotron Radiochemistry Facility, The University of Texas MD Anderson Cancer Center, Houston, TX 77054, USA

[4]RADIATE R&D Platform, The University of Texas MD Anderson Cancer Center, Houston, TX 77054, USA

[5]Department of Nuclear Medicine, The University of Texas MD Anderson Cancer Center, Houston, TX 77030, USA

[6]Department of Mechanical Engineering, Vanderbilt University, Nashville, Tennessee 37235, USA

*Email address: leon.bellan@vanderbilt.edu

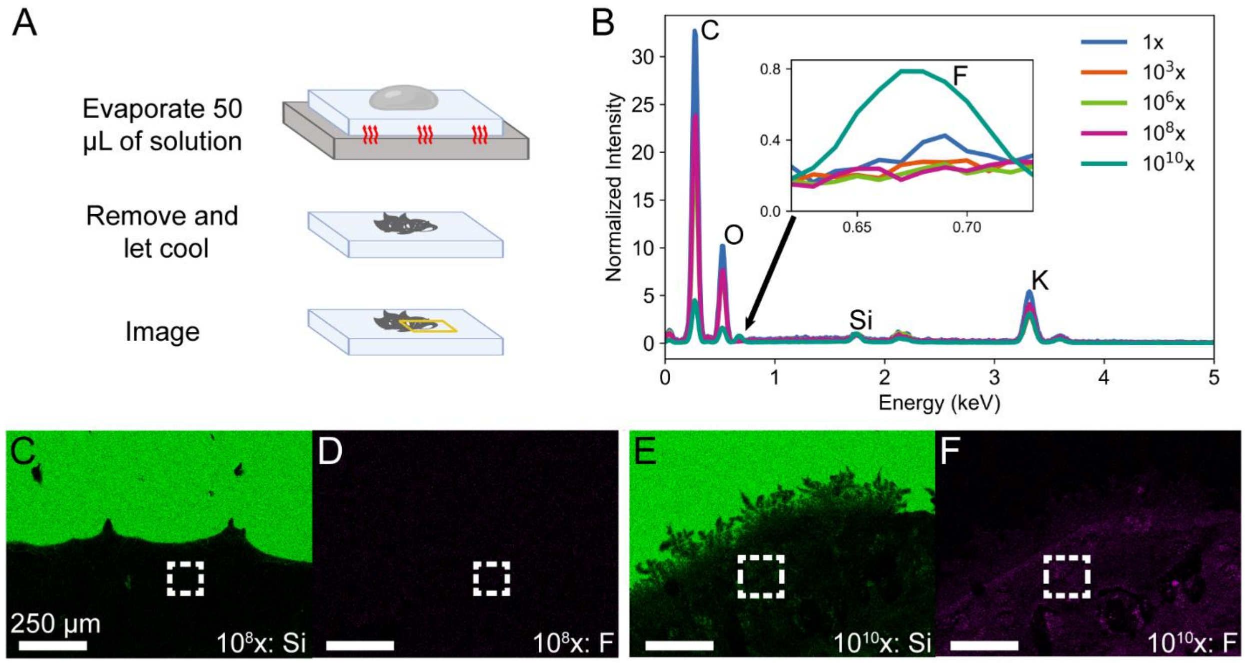

Figure S1. A) 50 μL solutions of various KF concentrations were evaporated from slabs of PDMS and analyzed using SEM-EDS. Solutions are labeled by the relative concentration of KF in the solution ranging from 1x (6.15pM) to 10^{10}x (61.5mM). $K_{2.2.2}$ and K_2CO_3 concentrations (26.2 mM and 21.7 mM, respectively) were kept constant. B) EDS spectra of salt deposits after evaporation; each spectrum was normalized to its Si peak. EDS images of C) Si and D) F from the salt deposits of 10^8x and E) Si and F) F from the salt deposits of 10^{10}x. The 10^{10}x solution was chosen as the main reaction solution for further analysis due to its appreciable F signal.

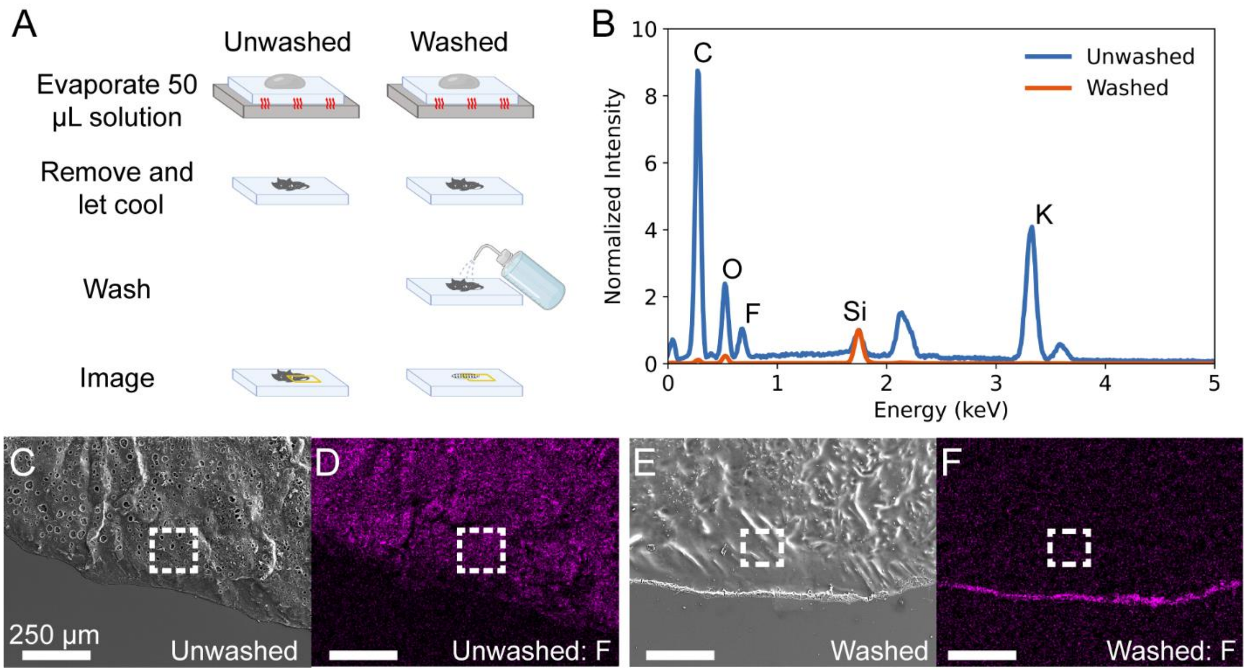

Figure S2. A) 50 µL solutions of 10^{10}x_F were evaporated on slabs of PDMS. One slab was not washed ("Unwashed") and the other was washed with 25 mL of ACN ("Washed"). B) EDS spectra of the salt deposits (or the area where the salt deposit was for the washed sample); each spectrum was normalized to its Si peak. The F peak is substantial for the unwashed sample but is undetectable for the washed sample, indicating complete removal of salt. C) SEM and D) EDS images of the unwashed sample showing significant amount of fluoride still present. Similar E) SEM and F) EDS images of the washed sample show complete removal of salt from the surface except for a small ring of salt likely formed from a coffee ring effect.

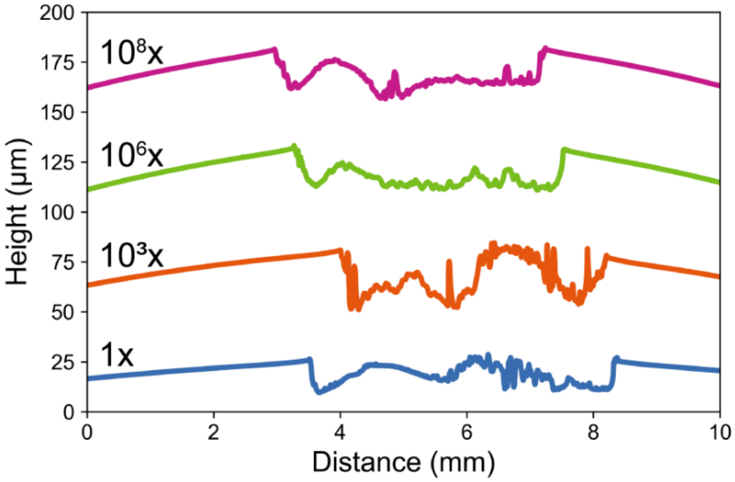

Figure S3. Profilometry results for solutions containing decreasing concentrations of KF. Samples were heated for a total of 15 min and otherwise prepared and tested in the same fashion as other profilometry experiments. As in S1, solutions are labeled by the relative concentration of KF.
